# Lumbar Vertebral Fracture Through a Pre-Existing Schmorl’s Node Mimicking Histopathologically a Low-Grade Chondrosarcoma

**DOI:** 10.7759/cureus.63468

**Published:** 2024-06-29

**Authors:** Kyriakos Papavasiliou, Thierry Lazure, Jessica Ghaouche, Charlie Bouthors, Charles Court

**Affiliations:** 1 Department of Orthopedic and Trauma Surgery, Spine and Tumor Surgery Unit, Hôpital Bicêtre, Assistance Publique Hôpitaux de Paris, Université Paris-Saclay, Paris, FRA; 2 Department of Pathology, Hôpital Bicêtre, Assistance Publique Hôpitaux de Paris, Université Paris-Saclay, Paris, FRA; 3 Department of Radiology, Hôpital Bicêtre, Assistance Publique Hôpitaux de Paris, Université Paris-Saclay, Paris, FRA

**Keywords:** spinal tumor, kyphoplasty, vertebral fracture, schmorl’s node, mid-grade chondrosarcoma, low-grade chondrosarcoma

## Abstract

The aim of this paper is to present a unique, to the best of our knowledge, case of a patient with a fracture of the first lumbar vertebra (L1), which occurred through a pre-existing Schmorl’s node (SN), with histopathological characteristics mimicking a low-grade chondrosarcoma that initially led to a false diagnosis. A 54-year-old woman tripped and fell to the ground, sustaining a fracture of the L1 vertebral body. She was treated conservatively with gradual mobilization using a thoracolumbar brace for six weeks. Due to persistent pain and her inability to achieve full mobilization, she was offered vertebral kyphoplasty. During the same operative session and just before the kyphoplasty, she underwent a core-needle biopsy of the affected area. Following her operation, she reported a gradual, yet quick and full remission of her symptoms. The pathology report indicated findings consistent with a low to mid-grade chondrosarcoma. A re-evaluation of the specimen by a different pathologist confirmed the diagnosis of low-grade chondrosarcoma. Subsequently, she underwent full oncological staging, which was negative for metastases. Additional imaging studies failed to show signs of local disease progression. Due to the discordance between the pathology reports and the imaging and clinical findings, her case was referred to our specialized center for spinal tumor surgery. A new pathological re-evaluation of the biopsy samples was performed, and the diagnosis of low-grade chondrosarcoma was once again confirmed. However, during the multidisciplinary tumor (MDT) meeting that followed, and after careful evaluation of subsequent imaging studies that showed signs of local improvement and due to the complete lack of symptoms, the histopathological findings were re-evaluated and attributed to the fracture occurring through a pre-existing SN penetrating the cancellous bone of the vertebra. This complex situation contributed to histopathological findings consistent with a well-differentiated chondrosarcoma. The patient remains symptom-free 10 months following her operation and has fully returned to her previous activities. Our unique case highlights the importance of an MDT meeting when evaluating patients with musculoskeletal tumors and emphasizes the need for increased awareness when clinical findings and imaging studies are in discordance with histopathology reports.

## Introduction

Sarcomas are tumors of mesenchymal origin that develop in the musculoskeletal system. They account for nearly 21% of all pediatric and for less than 1% of all adult solid malignant cancers [1]. Primary chondrosarcomas are sarcomas characterized by their ability of cartilage formation [2], with an incidence rate of 0.27-5.4 per million per year [3]. Although they can occur in both the axial and appendicular skeletons, the incidence of spinal chondrosarcomas accounts for only 2-12% of their total number [4]. The lumbar region is affected by approximately 20-33% of spinal chondrosarcomas [5].

A Schmorl’s node (SN) is typically defined as a vertical herniation of the nucleus pulposus through the cartilaginous and bony end plate into the body of an adjacent vertebra [6]. Its reported incidence ranges from 38% to as high as 79% [6]. Being often incidental findings in magnetic resonance imaging (MRI) studies [6], the clinical significance of an SN remains unclear [7].

The aim of this paper is to present the unique, to the best of our knowledge, case of a patient with a low-energy fracture of the first lumbar vertebra (L1), which occurred through a pre-existing SN, with histopathological characteristics similar to low-grade chondrosarcoma, initially leading to a misdiagnosis.

## Case presentation

**Figure 1:**
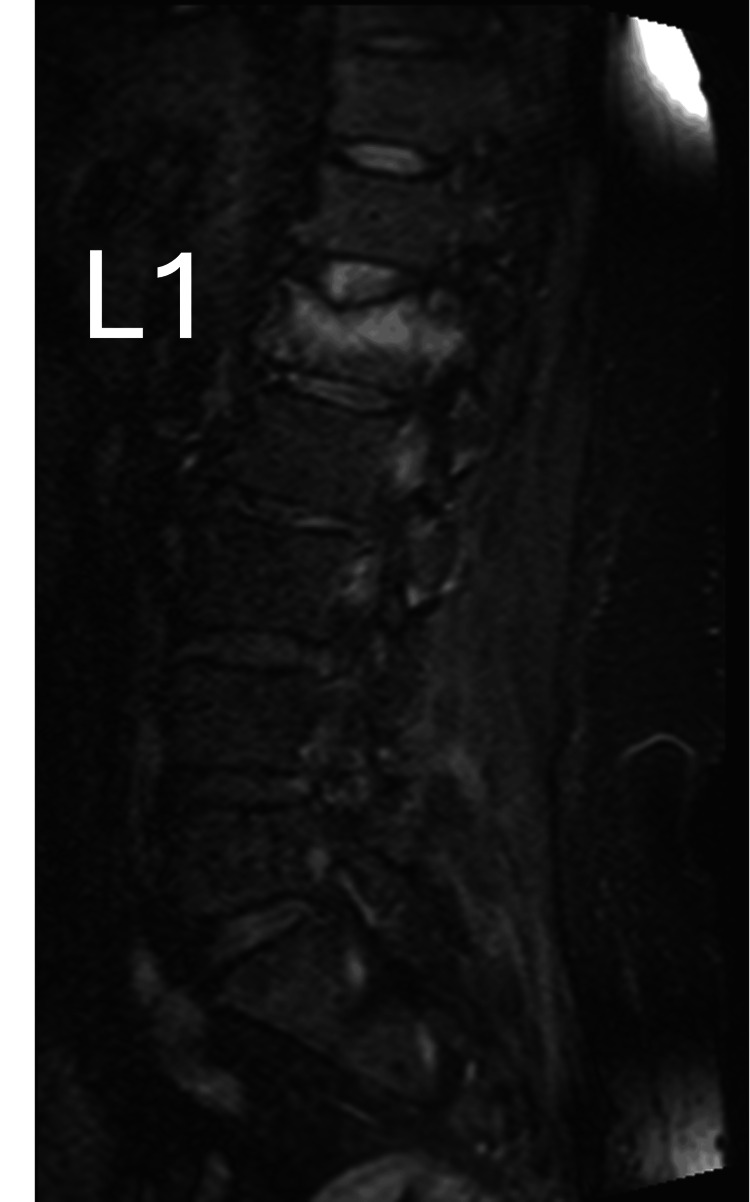
MRI TIRM T1 weighted image (sagittal view) of the lumbar spine obtained immediately following the fracture showing the edema of the vertebral body of L1. TIRM: turbo inversion recovery magnitude

A 54-year-old woman in good general condition, with no history of back pain, tripped and fell, sustaining a low-energy AO Spine type A1 fracture of the L1 vertebra [8], with no neurological deficit (Figure 1).

**Figure 2:**
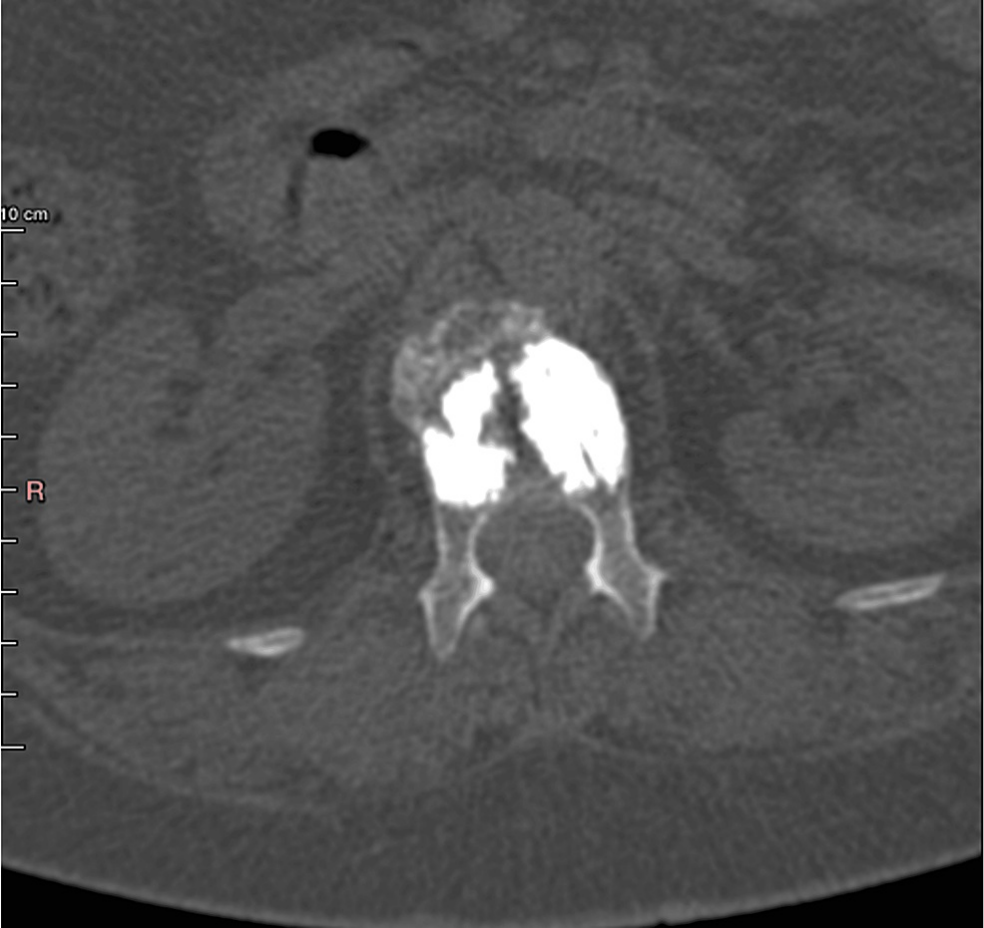
CT-scan (axial view) of the lumbar spine obtained immediately following the two balloons kyphoplasty performed on the L1. No gross signs of a malignant tumor and/or breaching of the vertebral cortex are apparent.

The patient received conservative treatment with a thoracolumbar brace for six weeks. Due to persistent pain and her inability to gain full mobilization, she was offered a vertebral kyphoplasty. Given her age, the absence of a prior osteoporosis diagnosis, and the low-energy trauma leading to her fracture, a core-needle biopsy of the affected vertebral area was performed during the same operative session, prior to the two-balloon kyphoplasty. The entire procedure went uneventfully, and she was discharged the next day, after undergoing a CT-scan confirming the accurate placement of the two balloons (Figure 2). Following her operation, she reported a gradual yet quick and full remission of her symptoms.

**Figure 3:**
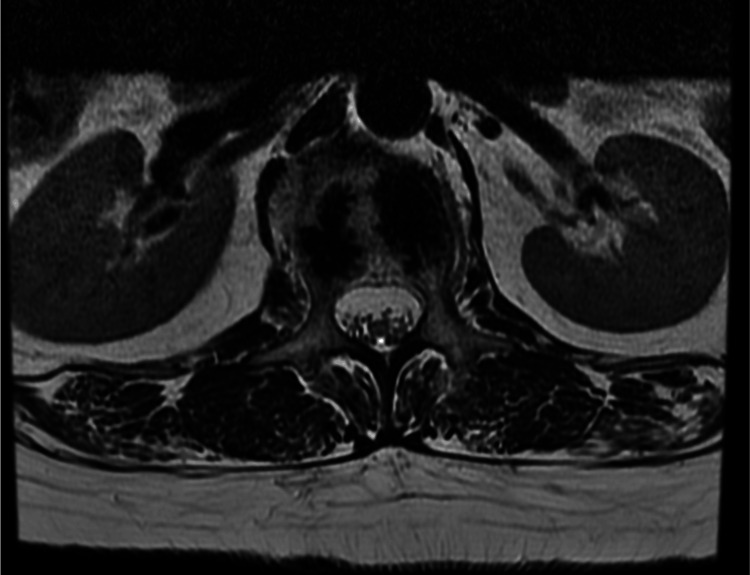
MRI T2 weighted image (axial view) of the lumbar spine obtained at three months following the fracture. No gross signs of a malignant tumor at the L1 vertebra, and/or breaching of the vertebral cortex are apparent.

The pathology report indicated findings consistent with a Grade I to Grade II chondrosarcoma [9]. All specimens were re-evaluated by a different pathologist, who confirmed the diagnosis of a Grade I chondrosarcoma. The patient then underwent full oncological staging, which was negative for metastases. In accordance with her clinical improvement, a new contrast-enhanced MRI scan of the lumbar spine was performed approximately three months after the fracture, which showed no signs of local disease progression (Figure 3).

**Figure 4:**
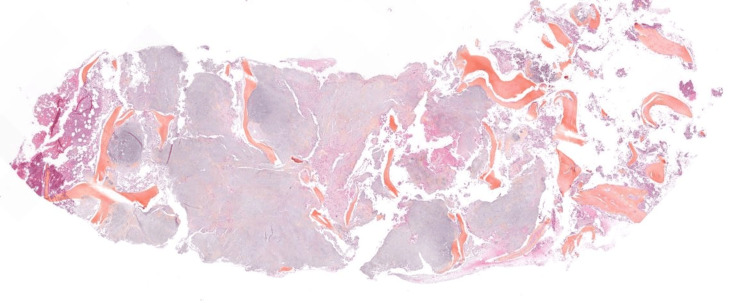
Microphotograph of the bioptic cylinder of the L1 vertebral body showing a chondroid lesion with degenerative change (HES; X12.5 magnification). HES: hematoxylin, eosin, and saffron

**Figure 5:**
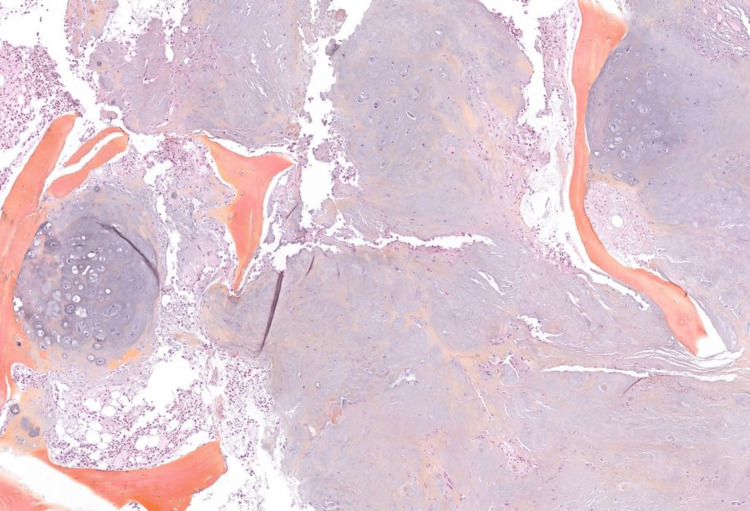
Microphotograph of the bioptic cylinder of the L1 vertebral body showing cancellous bone infiltrated by a very well-differentiated cartilaginous tumor (HES; X40 magnification). Chondrosarcoma-like lobules permeate between the vertebral bony trabeculae and fill the bone marrow spaces, surrounding the bony trabeculae of the cancellous bone and eroding them. The cellularity is low. Chondrocytes are dispersed within a hyaline matrix. They are discreetly atypical, with a spiculate, hyperchromatic nucleus. There is no binucleation or mitosis. HES: hematoxylin, eosin, and saffron

The patient was referred to our specialized center for spinal tumors surgery with the diagnosis of spinal chondrosarcoma. A new pathology re-evaluation of the biopsy samples was performed (Figure 4, Figure 5) and the diagnosis of a low-grade chondrosarcoma was once again confirmed.

**Figure 6:**
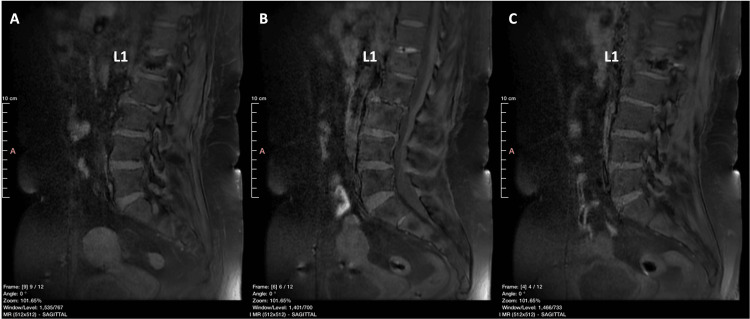
MRI fat suppression T1 weighted image (sagittal view) of the lumbar spine obtained at four months following the two balloons kyphoplasty. No gross signs of a malignant tumor at the L1 vertebra, and/or breaching of the vertebral cortex are apparent. A: left part of the spinal column, B: medial part of the spinal column, C: right part of the spinal column

**Figure 7:**
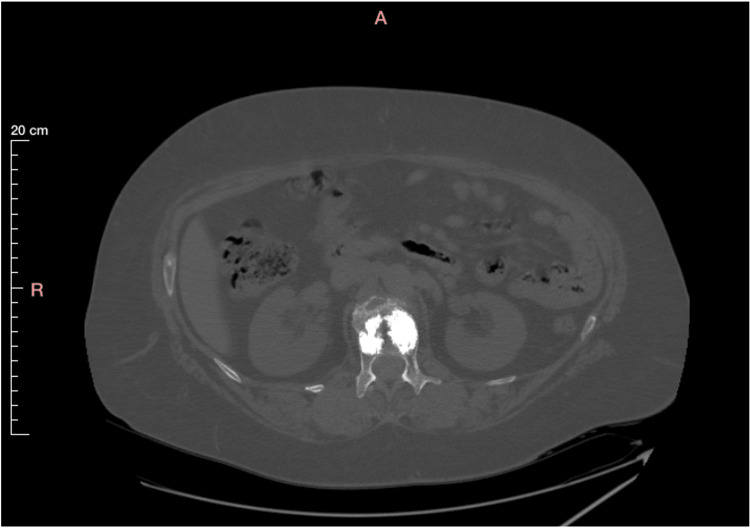
CT scan (axial view) of the lumbar spine obtained at four months following the two balloons kyphoplasty. No gross signs of a malignant tumor at the L1 vertebra, and/or breaching of the vertebral cortex are apparent.

During a multidisciplinary tumor (MDT) meeting, which included tumor spine surgeons, a musculoskeletal radiologist, and a pathologist, existing and newly acquired imaging studies from an MRI scan (Figure 6) and a CT scan (Figure 7) were carefully evaluated, showing signs of local improvement instead of disease progression. Furthermore, the patient remained symptom-free. The MDT meeting proposed that all specimens be re-evaluated with this new information in mind.

**Figure 8:**
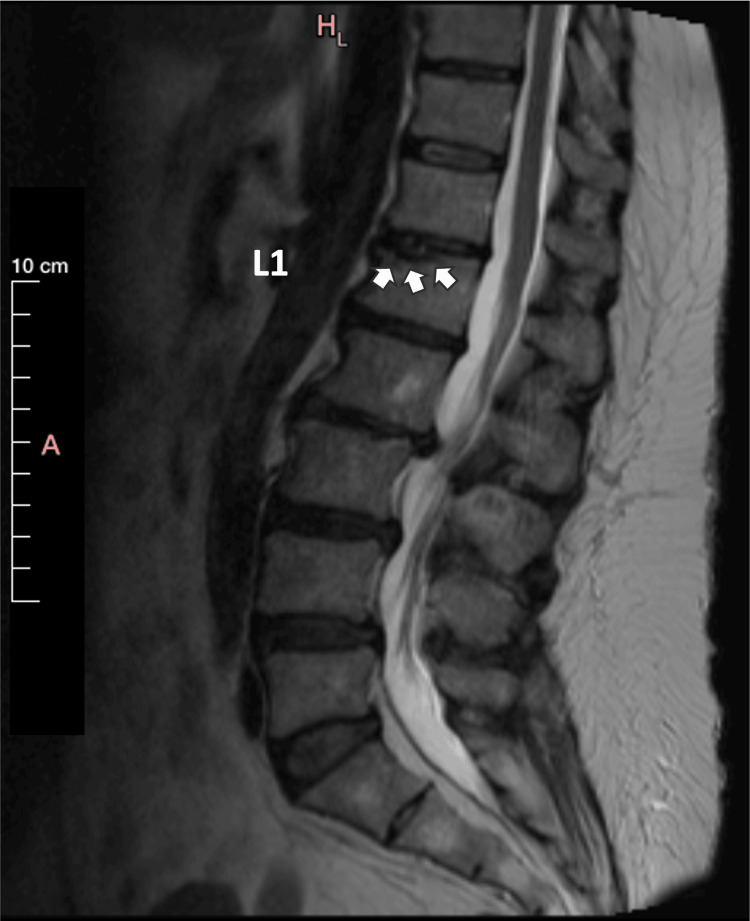
MRI TIRM T1 weighted image (sagittal view) of the lumbar spine obtained immediately following the fracture revisited. A Schmorl’s nodule is highlighted by the small white arrows. TIRM: turbo inversion recovery magnitude

**Figure 9:**
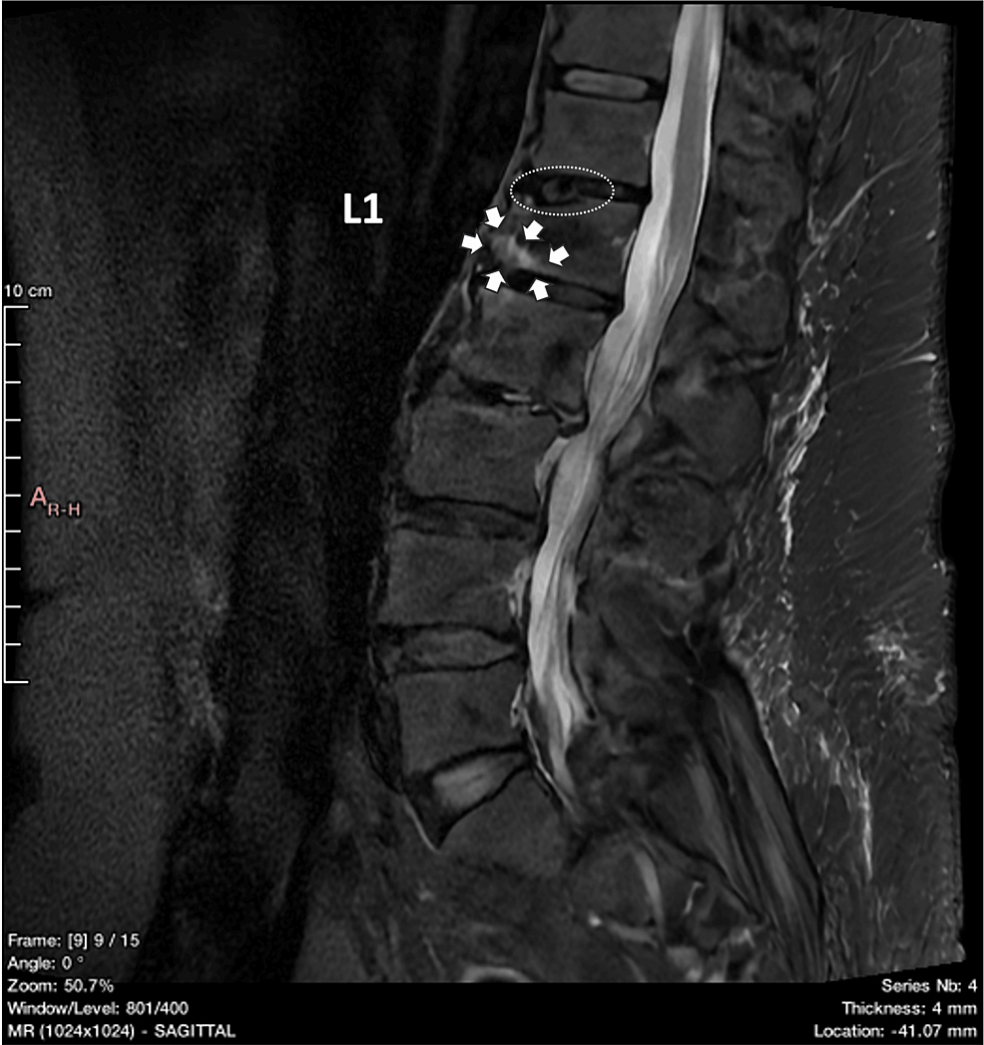
MRI AIR™ Recon DL STIR weighted image (sagittal view) of the lumbar spine obtained at four months following the two balloons kyphoplasty. A Schmorl’s nodule is highlighted by the white dotted oval-shaped line. The small white arrows point at the remaining edema. No gross signs of a malignant tumor at the L1 vertebra, and/or breaching of the vertebral cortex are apparent. AIR™ Recon DL, GE HealthCare Technologies, Inc., Chicago, Illinois, United States STIR: short tau inversion recovery

This final evaluation concluded that all histopathological findings should be attributed to the vertebral fracture occurring through a pre-existing SN. Penetration of the fibrocartilage of the SN through the fracture of the vertebral plateau depicted cartilage within the cancellous bone. This was previously misinterpreted as chondrosarcoma, as the pathologist had not reviewed the imaging studies showing the penetration of the cancellous bone of the vertebra by the SN, as depicted in the MRI scan performed immediately after the fracture (Figure 8). This same penetration was also obvious in an MRI scan performed at four months after the kyphoplasty (Figure 9).

**Figure 10:**
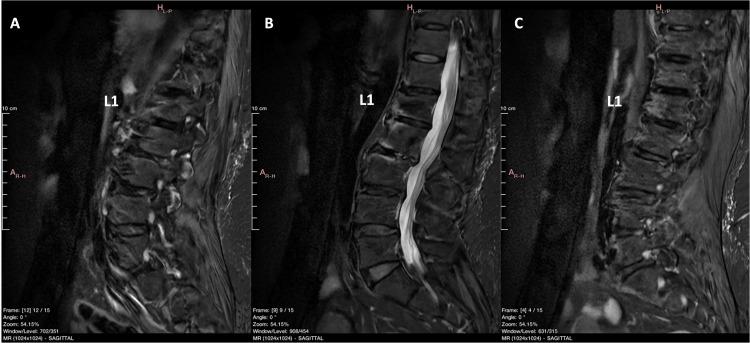
MRI STIR FSE images (sagittal view) of the lumbar spine obtained at 10 months following the two balloons kyphoplasty. No gross signs of a malignant tumor at the L1 vertebra, and/or breaching of the vertebral cortex are apparent. A: left part of the spinal column, B: medial part of the spinal column, C: right part of the spinal column STIR: short tau inversion recovery; FSE: fast spin echo

This complex situation contributed to histopathological findings consistent with, yet mimicking, a well-differentiated chondrosarcoma [10]. The patient remains symptom-free at 10 months following her operation. Her most recent imaging studies (Figure 10) show no signs of neoplastic disease, and she has fully returned to her previous activities.

## Discussion

Our unique case of a patient with a fracture of L1 sustained through a pre-existing SN, initially misdiagnosed as low-grade chondrosarcoma, highlights the importance of an MDT meeting when evaluating patients with musculoskeletal tumors.

First described in 1927 by the pathologist Christian Georg Schmorl, SNs are typically defined as intravertebral disc herniations [6]. They are commonly found in the lumbar spine and are usually noted as endplate irregularities, abnormalities, or defects [7]. Various etiologies for their development have been proposed, including traumatic, idiopathic, developmental, genetic, and decreased bone mineral density factors. Most importantly, their clinical relevance remains uncertain, largely due to variations in the definition of SN, population characteristics, assessment methods, and sample sizes. SNs are often associated with degenerative lumbar disease [7]. The latter was certainly not the case in our patient.

Chondrosarcomas usually appear de novo in the musculoskeletal system, although patients with benign cartilaginous tumors such as osteochondromas, and especially with multiple enchondromas [11], have an increased risk for malignant transformation of their benign lesions [12]. Unlike most spinal tumors, chondrosarcomas rarely (5% of cases) arise in the body of a vertebra, as the majority are found either in its posterior parts or in both the anterior and posterior vertebral elements [4]. The most common symptom of chondrosarcoma is pain, while a palpable mass and/or a neurologic deficit can be found in 50% of patients [13]. A pathological fracture is seldom, if ever, the first manifestation of an otherwise occult vertebral chondrosarcoma. Furthermore, the majority of A1 AO Spine pathological vertebral fractures are osteoporotic [8]. Metastatic lesions may also cause a pathological fracture, even though most of them usually arise in the posterior elements of the vertebral body [14]. These facts certainly raised suspicion regarding the accuracy of the initial histopathological diagnosis in our patient, considering the low-energy trauma and the non-osteoporotic spine.

Chondrosarcomas are characterized by their notorious resistance to neo-adjuvant and/or adjuvant conventional chemotherapy [4]. Regarding radiotherapy, a very high effective dose of 66-70 Gy for microscopically positive margins and a dose of 70 Gy for gross residual disease are necessary [2]. Conventional radiotherapy with lower doses results in high local recurrence rates. As a result, chondrosarcoma remains a “surgical disease”. An R0 “en-bloc resection” is the only viable therapeutic option for patients suffering from such a tumor [15], even though recent advances with proton beam therapy attempt to avoid exceeding adjacent tissue tolerances and show promising results when combined with surgery [16].

Surgery should aim to preserve, if not improve, functionality, relieve pain, offer local disease control and prolonged survival, and prevent recurrence [17]. In our case, if the original diagnosis of vertebral chondrosarcoma had been confirmed, a complete 360° vertebrectomy of L1, with potential resection of the L1 and/or T12 nerve roots (depending on the exact location of the tumor) and a complex reconstruction with a cage and spinal fusion would have been necessary [18-20]. This complex procedure, which is accompanied by increased morbidity, would have been the only treatment option providing a realistic chance to achieve R0 resection of both the tumor located at the body of the vertebra and the surgical tracks from the previously performed kyphoplasty. Depending on the resection margins, adjuvant radiotherapy could also have been necessary. 

## Conclusions

This case highlights the importance of an MDT meeting when evaluating patients with musculoskeletal tumors, especially when clinical findings, imaging studies, and pathology reports are in discordance. As demonstrated in this case, expertise through knowledge and research, experience gained from practice, careful evaluation of clinical data and imaging studies, and discussion between physicians of different specialties, lead to proper diagnosis and correct decisions regarding the treatment of patients with these rare diseases.
